# Outbreak of eczema and rhinitis in a group of office workers in Greenland

**DOI:** 10.3402/ijch.v74.27919

**Published:** 2015-07-01

**Authors:** Niels E. Ebbehøj, Tove Agner, Erik Zimerson, Magnus Bruze

**Affiliations:** 1 Department of Occupational and Environmental Medicine, Bispebjerg Hospital, University of Copenhagen, Copenhagen, Denmark; 2 Department of Dermatology, Bispebjerg Hospital, University of Copenhagen, Copenhagen, Denmark; 3 Department of Occupational and Environmental Dermatology, Skane University Hospital, Lund University, Malmø, Sweden

**Keywords:** work place study, eczema, urticarial, rhinitis, patch test, indoor climate

## Abstract

**Introduction:**

Disturbed indoor climate may in some cases be associated with illness. In the present paper, we report the results from a thorough investigation of office workers in Greenland, who developed skin and/or airway problems after moving into renewed offices.

**Material and methods:**

In 2009 the office of the Bank of Greenland had a total renovation of the building, including new furniture and carpets. Symptoms developed within the first year after moving back into the renewed buildings. After removal of carpets in the building, symptoms significantly improved. Workers were examined in 2009 and re-examined in 2013, including clinical examination, patch test and when relevant also skin prick tests and histamine release test. Isothiazolinones and fumarates, both able to cause airway as well as skin symptoms, were isolated from carpets before testing, and included in the test series.

**Results:**

In total, 32 out of 80 workers (40%) developed symptoms; 27 reported eczema, 20 rhinitis and 4 urticaria. Eczema was located on the hands and/or lower arms in 18 workers, on the face in 10 workers and on legs/trunk in 12 workers. After intervention in the office, 22 workers with eczema reported significant improvement, all cases of hand eczema cleared and 16 workers with rhinitis also improved. Positive patch tests to carpet extracts were found significantly more frequent in the worker cohort than in a control group comprising 47 dermatitis patients (p<0.001). Only few workers reacted with a positive response to skin prick test or in the HR test, no obvious pattern in reactions was found, and no conclusions can be made from these reactions.

**Conclusion:**

The results indicate that the reported symptoms are related to exposures from the building after renovation in 2009. A specific triggering exposure could not be identified, although chemicals from the glued carpets are suspected. The study is an example of a work place investigation, and illustrates the diversity of symptoms and exposures involved in “Sick Building” cases.

Disturbed indoor climate may in some cases be associated with illness occurring in workers at the work place site (1). Prevalence of “Sick Building Symptoms” has been related to indoor air quality, psychosocial conditions and personal factors such as atopy and photosensitive skin (2). Facial skin irritation, eye irritation and nasal symptoms are frequently reported symptoms, as well as more general symptoms such as fatigue and headaches (3). Prevalence of seborrhoic dermatitis has been related to the use of visual display terminals (4), and physical irritant contact dermatitis has been related to low indoor humidity due to air-conditioning (5). Fungal and house dust mite aeroallergens may contribute to rhinitis symptoms (6), and airborne contact allergens in newly renovated buildings may be involved as well (7). However, although allergy testing may contribute to a clarification, it is sometimes difficult to obtain a specific explanation. Workplace visits are important to fully understand exposure and workflow, but even after thorough examination of exposures the situation may sometimes remain difficult to solve.

In the present paper, we report the results from a thorough investigation of office workers, who developed skin and/or airway problems after moving into new premises. During the period 2009–2012, similar complaints were reported from 3 different workplaces: 1 in Greenland (a bank office) and 2 in Denmark (a bank office and a telecom office). Complaints were eczema, rhinitis and urticaria. In all 3 worksites, the buildings/offices had been renewed, including new carpets in the offices, which were identical for the 3 workplaces. We here report exposures, prevalence of symptoms, results of allergy tests and follow-up data from workers in one of these worksites (the Bank of Greenland), and an interpretation of the situation is attempted.

## Material and methods

### Workers

In Nuuk, Greenland, the office of the Bank of Greenland had a total renovation of the building, which was finished in 2009. Staff were moved out of the building while the restoration took place, and moved back into the building when it was finished. New carpets as well as new furniture had been provided for most office rooms at the workplace. In March 2009, a total of 80 office workers moved back into the renovated premises, and in September 2009, the symptoms began. The complaints were mainly skin problems (eczema, and less frequent urticaria) and rhinitis. All workers (n=32) complaining of any of these symptoms were referred by the employer and examined in 2009–2010. A history of atopic dermatitis according to the UK criteria (8) and a history of rhinitis were recorded. A clinical examination was performed verifying skin symptoms, and in patients with eczema a patch test with TRUE test was performed. Persons with eczema were working in all parts of the building, except for a minor area with wooden floor and no carpets. Two persons with severe eczema were moved into this part of the building, and readily improved significantly, while symptoms continued in workers who had not been moved into carpet-free rooms. All carpets were changed into new, but identical carpets without glue, but symptoms continued in most workers. Consequently, it was decided in 2011 to remove all carpets from the renewed building. This resulted in an improvement in most of the workers. However, a small group of workers still had symptoms (see below). Since the desks in the offices were made of a soft rubber material attracting dust to the surface of the desks, it was suspected that these desks could also be part of the problem, and following the removal of the carpets, the desks were now changed into new ones with a smooth surface, in the autumn 2012. After these significant changes in the building had been performed (see below), all workers were re-examined in February 2013. The re-examination included a clinical examination and a patch test with allergens identified in the carpet and the carpet glue offered to all workers with previous or current symptoms. High-performance liquid chromatography (HPLC) and gas chromatography-mass spectrometry (GCMS) were used for identification of allergens in the carpets. Analyses were performed at the laboratory in Malmö. The analytic procedure is described in detail elsewhere (9). The carpets in the renovated Bank of Greenland were examined (with and without glue), and carpets from one of the other workplaces, where workers had had similar complaints (the telecom office) were also examined. The carpets from Bank of Greenland were Interface Floor^®^ The Scandinavian Collection Helsinki, batch no. 308031005/303101. The carpet glue was PCI Teppefixering^®^ FRS 387 from BASF. The material safety data sheet did not provide information of its content.

### Patch test

In 2009–2010, patients with skin symptoms were tested using TRUE test (series 1 and 2, a commercial available standard series for patch testing). Patches were left on for 48 hours, and reading was performed at Day 2 or 3. In 2013, a patch test series was composed based on the results of the chemical analyses, consisting of the carpet extracts and some single allergens which were identified and judged to be possibly relevant. The patch test series applied in 2013 is shown in Table I. Patch testing was performed using 8 mm Finn Chambers, left on for 48 hours. Readings were performed at Day 3, and a trained dermatological nurse assessed response. All patients were informed to report any delayed reactions. Controls were recruited in 2013 from dermatitis patients from the Department of Occupational and Environmental Dermatology, Malmø. Controls were patch tested with Carpet 1 100% extract (with glue) from Bank of Greenland, and with Carpet 4 100% extract.

**Table I:** Number of patients with positive patch tests (+ or ++) to carpet extracts and identified allergens

	Total patients (n=31)	Eczema (n=26)	Urticaria or rhinitis only no eczema (n=5)	Control group (n=47)
Carpet 1 100%	0	0	0	–
Carpet 1 10%	1	1	0	–
Carpet 1 with glue 100%	10	8	2	2
Carpet 1 with glue 10%	0	0	0	–
Carpet 2 100%	0	0	0	–
Carpet 2 10%	0	0	0	–
Carpet 3 100%	1	1	0	–
Carpet 3 10%	0	0	0	–
Carpet 4 100%	5	4	1	1
Carpet 4 10%	0	0	0	–
BIT 0.05% pet	0	0	0	–
MI/MCI 0.020% aq	0	0	0	–
MI 0.20% aq	0	0	0	–
DEHF 5.0% pet	0	0	0	–
DEHF 0.50% pet	0	0	0	–
Dimethyl fumarate 0.10% pet	1	1	0	–

Results shown for all participants, for patients with eczema symptoms, for patients with hand eczema (HE), for patients with other eczema only, and for patients with other eczema localisation only. Carpet 1: From Bank of Greenland, tested with and without glue (ethanol extract). Carpet 2: From Bank of Greenland (ethanol extract). Carpet 3: From telecom workplace, location 1 (ethanol extract). Carpet 4: From telecom workplace, location 2 (ethanol extract). BIT: 1,2-Benzisothiazol-3(2H)-one; MI/MCI: 2-methyl-4-isothiazoline-3-one and 5-chloro-2-methyl-4-isothiazoline-3-one (Kathon CG); MI: 2-methyl-4-isothiazoline-3-one; DEHF: bis(2-ethylhexyl) fumarate.

### Type 1 allergy

All workers were exposed to 15 microliter extract from carpets (same extract as used for patch test, see above) from the Bank of Greenland (6 extracts in total) as open test for 20 minutes. Those who reacted with a flare went on to have a prick test with the specific extract that they had reacted to. Blood samples for histamine release test (HR test) were taken and analyzed towards the same extracts as used for the patch tests (10, 11, 12).

## Results

### Investigations 2009–2010

In total, 32 (10 men and 22 women) out of 80 workers (49 women and 31 men; median age 51.5 years) reported work-related symptoms after moving back into the renewed buildings in 2009. No significant difference was found between workers who did and who did not develop symptoms with respect to age or sex distribution. Previous or current atopic eczema was reported by 2 workers, and 2 reported psoriasis. Twenty-seven workers reported onset of eczema, 20 workers reported onset of rhinitis and 4 reported urticarial, all with onset in the period March 2009–February 2010. Eczema was located on the hands and/or lower arm in 18 workers, face in 10 workers and legs/trunk in 12 workers. Location at the hands and arms were in most cases at the ulnar side of the hand and/or arm, and at the hypothenar area of the palms. Nine workers only had eczema symptoms outside the hands.

#### Allergy testing 2009–2010

Of the 27 workers identified with eczema in 2009–2010, 26 underwent patch testing. Nineteen had negative response. Seven responded with positive patch tests: 4 reactions to nickel, 2 to thiuram, 2 to quinolone, 1 to chromate, 1 to PPD and 1 to *p*-*tert*-butyl-phenol-formaldehyde resin. None of these reactions were considered relevant with respect to the current eczema.

#### Other investigations from the work place

The relative humidity as measured in the work place after renovation in 2010 was measured as <10% during wintertime because of the very cold and dry outdoor climate. Measures were taken to increase indoor humidity and later measurements from 2011 were registered as 20–30%. An investigation for mould was negative. Workers in the Bank of Greenland were generally satisfied with their working situation, and in Nuuk the Bank of Greenland office is generally looked upon as a privileged work place.

### Investigations 2013 (after removal of carpets)

In 2013, all workers who originally complained of eczema, rhinitis or urticaria were re-examined. Five had left their job since first investigation, but still participated in the re-examination.

A total of 22 workers with eczema reported significant improvement after removal of carpets and change of desks, and 5 workers did not improve after changes of carpets and desks. In those with persistent skin symptoms, face-eczema in 2 workers and leg-eczema in 2 other workers could be verified at the clinical examination, while the persistent skin symptoms on the hand in 1 worker turned out to be dermatomycosis, and trichophyton rubrum was isolated. With respect to rhinitis, 18 workers out of 20 reporting rhinitis improved after removal of carpets and change of desks.

#### Chemical analyses

The results of the HPLC analysis are given in Table II. BIT and MI were detected in Carpet 2.

**Table II:** Results from HPLC analysis of the presence of 1,2-benzisothiazol-3(2H)-one (BIT), 2-methyl-4-isothiazolin-3-one (MI), and 5-chloro-2-methyl-4-isothiazolin-3-one (MCI) in the carpets

	Concentrations in the carpets			Concentrations in the 100% extracts		
		
Sample	BIT µg/cm^2^	MI µg/cm^2^	MCI µg/cm^2^	BIT µg/ml^a^	MI µg/ml^a^	MCI µg/ml^a^
Carpet 1	<0.03	<0.01	<0.001	<1	<0.4	<0.04
Carpet 1 with glue	<*0.03*	<*0.001*	<*0.008*	<*1*	<*0.04*	<*0.3*
Carpet 2	0.20	0.04	<0.001	7.3	1.5	<0.04
Carpet 3	<0.02	<0.02	<0.004	<0.8	<0.6	<0.15
Carpet 4	<*0.1*	<*0.04*	<*0.001*	<*4*	<*1.5*	<*0.04*

a µg/ml ≈ppm (1 ppm=0.0001%). Columns with carpet extracts to which positive patch test reactions were found are in italic.

Results from the GCMS analysis of the *carpet extracts* are given in Table III, in which major components are presented with determined or estimated concentrations. Apart from these, component *N*-methyl-1,2-benzisothiazol-3(2H)one-1,1-dioxid (CAS 15448-99-4) was detected only in Carpet 1 with glue and in Carpet 4.

**Table III:** Major components detected in the carpets and their concentrations in the patch test extracts

Substance	Carpet 1 mg/ml	Carpet 1 with glue mg/ml	Carpet 2 mg/ml	Carpet 3 mg/ml	Carpet 4 mg/ml
Bis(2-ethylhexyl) fumarate^a^ CAS 141-02-6; MW 340	4.26	*6.12*	3.41	7.35	*4.15*
Bis(2-ethylhexyl) maleate^a^ CAS 142-16-5; MW 340	0.32	*0.58*	0.50	0.57	*0.08*
*N*-Methyl-1,2-benzisothiazol-3(2H)-one^b^ CAS 2527-66-4; MW 165	0.01	*2.2*	<0.01	1.9	*2.2*
*N*-Methyl-1,2-benzisothiazol-3(2H)-thione^b^ CAS 15871-24-6; MW 181	0.01	*0.81*	<0.01	1.4	*0.46*
1,8-Diazacyclotetradecane-2,7-dione^c^ CAS 4266-66-4; MW 226	1.1	*0.6*	1.1	0.44	*0.21*
Caprolactam^c^ CAS 105-60-2; MW 113	<0.003	*0.028*	0.29	0.006	*0.009*

a Bis(2-ethylhexyl) fumarate used as reference substance for determination/estimation of concentrations.

b 1,2-Benzisothiazol-3(2H)-one used as reference substance for estimation of concentrations.

c Caprolactam used as reference substance for determination/estimation of concentrations.

Columns with carpets extracts to which positive patch test reaction were found are in italic.

#### Allergy testing

Of the 32 workers initially reporting work-related symptoms in 2009–2010, 31 participated in allergy testing, and one did not participate due to pregnancy.

#### Results of patch testing

Patch test results can be seen in Table I. Twelve workers responded to one or more allergens in the test series with a positive reaction, while 19 had negative outcome to the test. A total of 10 workers (32%) had a positive reaction to Carpet 1 100% extract (with glue) from Bank of Greenland. Five workers reacted positively to patch testing with Carpet 4 100% extract, and of these 4 had a concomitant positive reaction to Carpet 1 100% with glue.

With respect to reactions to allergens other than the extracts only 1 positive reaction was found. This reaction was to dimethyl fumarate in a worker with hand eczema, and she also reacted positive to Carpet 1. No present or previous exposure to dimethyl fumarate was identified, and she had never had shoe dermatitis. No late reactions were reported by any of the tested workers.

#### Control group

Forty-seven patients recruited from dermatitis patients referred for patch testing at Department of Occupational and Environmental Dermatology, Skane University Hospital, Lund University, Malmø, Sweden, served as controls. In the control group, >30% of the patients had atopic dermatitis, which is significantly more than the 6.5% of the worker group (2 out of 31 tested workers).

Two had a positive reaction to Carpet 1, and 1 control patient had a positive reaction to Carpet 4. Significantly more workers than dermatitis patients reacted positively to testing with carpet extracts (p=0.01 and p=0.034 for Carpet 1 and Carpet 4, respectively).

#### Results of type 1 allergy testing

The results from type 1 allergy testing are given in Table IV. Of the 4 workers reacting with a flare to open test with carpet extracts, 2 of these had urticaria.

**Table IV:** Type 1 allergy tests 2013

Open application and patch test	HR test to carpet extracts
31 workers* were tested with open application of carpet extracts	19 workers with urticaria or rhinitis symptoms were examined with HR test**
4 of these reacted with a positive flare and went on to prick test	
2 of these reacted positive to carpet extracts from Bank of Greenland	

* All workers except one who was pregnant.

** 4 of these had a positive HR test to carpet extracts, 2 to carpet extracts from Bank of Greenland and 2 to carpet extracts from TDC; No overlap between the 4 workers responding to open application and the 4 workers with positive HR test.

#### Biopsy

A biopsy was taken from a positive patch test to Carpet 1 100% with glue when reading was performed at Day 3. Histology showed spongiosis and eczema, supporting an allergic reaction.

## Discussion

Outbreak of various symptoms in workers after renovation of office buildings is a problem which is not unusual, and a solution of the problem is not always simple. Here we present an example of a thorough investigation of skin symptoms in office workers with onset after building restoration. In the present investigation, a total of 32 out of 80 workers (40%) in the Bank of Greenland developed skin problems and/or rhinitis when moving into renovated premises. Following intervention in the building, 22 out of 27 initially complaining of eczema had improved, and all cases of hand eczema had cleared. Three out of four workers with urticaria had likewise improved. The onset of symptoms in relation to renovation of the building, the specific complaints, and the disappearance of symptoms after intervention at the workplace strongly indicates a relationship between exposures related to the renovated building and skin symptoms/rhinitis. Simultaneous outbreak of similar symptoms in workers from 3 different renovated offices, where the same carpets had been installed, further supports a link between worksite exposures and symptoms. Socio-psychological factors are less likely to play any important role in the present outbreak of skin symptoms and rhinitis in the Bank of Greenland, since it is generally accepted as an exclusive and attractive workplace in an area of the world where such workplaces are few.

In general, women are more prone to develop “Sick Building Symptoms” than men (13), and previous atopic disease is also a risk factor. Atopic dermatitis is known as a risk factor for development of irritant contact dermatitis. In the present study, no difference in sex distribution between workers with and without symptoms was found. Only 2 persons reported past or present atopic dermatitis, and this can therefore be ignored as explanation for the present findings. Since the carpets were identified as a factor in common for 3 different work places with onset of skin symptoms in office workers, and due to the fact that workers in the Bank of Greenland who were moved into carpets-free areas in the building became symptom-free, focus was directed at the carpet as a culprit. A comprehensive chemical investigation of the carpets and carpet glue was performed, which both clarified some questions but also put forward new ones. Caprolactam and 1,8-diazacyclotetradecane-2,7-dione, found in the carpet extracts, are used or can be formed during the production of polyamide (Table III). These substances show that polyamide was a major component in the carpets. However, none of these substances can be expected to be a potent allergen or irritating substance, and the highest concentration of these substances was found in Carpet 2, to which there was no positive patch test reactions (Table I).

**Fig. 1:**
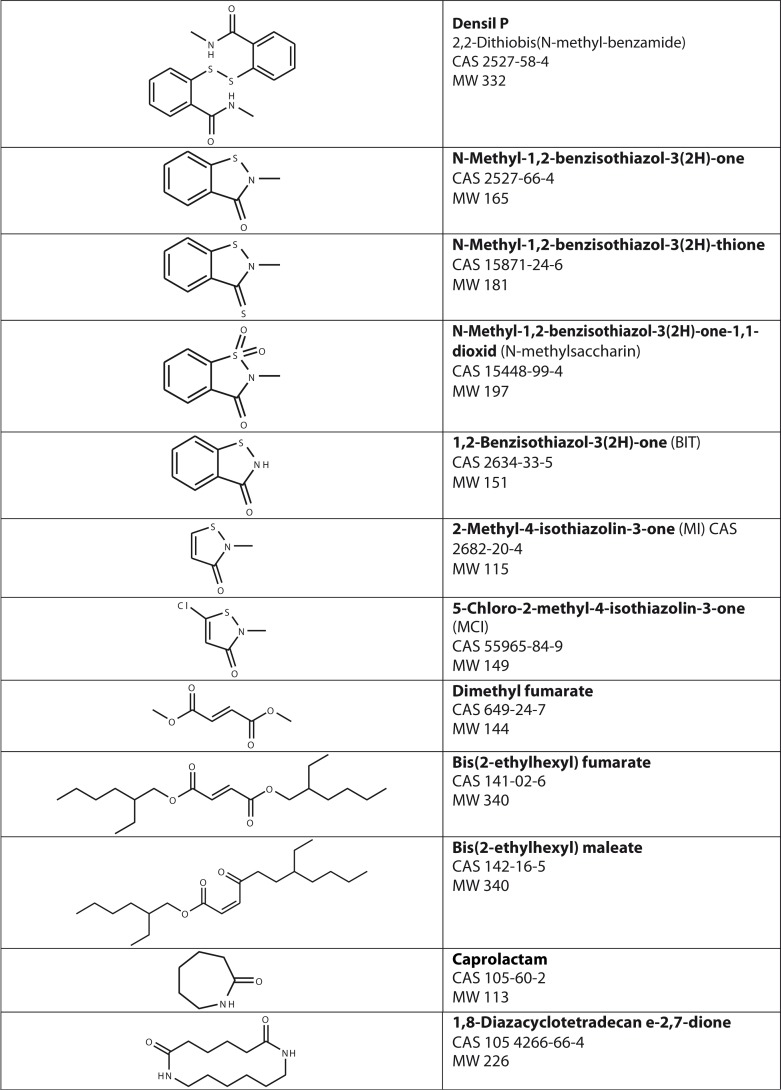
Chemical structures, CAS no and molecular weight for selected substances.

*N*-Methyl-1,2-benzisothiazol-3(2H)-one and *N*-methyl-1,2-benzisothiazol-3(2H)-thione were also both found in relatively high amounts in Carpet 1 with glue, as well as in Carpet 3 and 4. We had the information that some of the carpets could have been treated with an antimicrobial agent Densil P (Fig. 1). We did not detect Densil P in any of the carpet extracts but instead the 2 closely related substances above. It is possible that Densil P could form these substances by thermal decomposition during the GCMS analysis, when the sample is heated to 250–300°C. We could not test this hypothesis as Densil P was not available on the market. Both *N*-methyl-1,2-benzisothiazol-3(2H)-one and *N*-methyl-1,2-benzisothiazol-3(2H)-thione were present in Carpet 1 and 4, but also in Carpet 3, to which no positive patch test reactions were seen. *N*-Methyl-1,2-benzisothiazol-3(2H)-one-1,1-dioxid, also known as *N*-methylsaccharin, was detected only in the carpet extracts giving positive patch test reactions, the extracts of Carpet 1 with glue and Carpet 4. *N*-Methyl-1,2-benzisothiazol-3(2H)-one-1,1-dioxid was not included in the patch test series.

The main component in the carpet extracts was bis(2-ethylhexyl) fumarate. Other esters of fumaric acid are known to be contact allergens. However, in the present study bis(2-ethylhexyl) fumarate cannot explain the exclusive positive reactions to Carpet 1 with glue and Carpet 4, because all extracts contained similar amounts. Bis(2-ethylhexyl) fumarate was also included in the patch test series but gave completely negative results although it was tested in a concentration approximately 10 times higher than in the 100% carpet extracts (14).

BIT and MI were detected in Carpet 2, and MI and MCI were detected in the carpet glue. Patch testing with these substances was, however, negative in all tested workers.

Indoor humidity was reported as being very low immediately after workers had moved back to the new premises, and increased after measures had been taken to change this. Low humidity is a well-known risk factor for development of skin symptoms (5); however, low humidity is a general problem indoor in Greenland due to cold and dry air to be warmed up indoor. Dry air may enhance the severity and frequency of eczema problems, but it is a less obvious explanation in the present setting.

With respect to allergy test for type 1 allergy, neither the few positive prick tests nor the few positive HR-tests contribute to a clarification. Furthermore, no overlap was found between positive prick and HR-tests, making it even less likely that the few positive results have any clinical relevance.

Thirty-one percent of workers with complaints reacted with a positive patch test to Carpet 1 (with glue) applied in the Bank of Greenland, and 16% reacted positive to Carpet 4. Number of positive allergic reactions was statistically significantly higher than that found in the control group. A higher frequency of irritant test reactions could be expected in the control group (dermatitis patients) than in the workers, since in the worker cohort only 6.5% reported atopic disease, as compared to more than 30% in the control group. This supports the interpretation of patch test results being true allergic reactions in the workers, and not due to irritancy, since the number of positive reactions in the control group would then be expected to be high. On the other hand, dry winter weather may also cause sensitive skin, and patch testing was performed during the autumn in the control group as compared to February in Greenland in the workers. Increased skin reactivity due to atopic dermatitis as well as time of the year may have influenced patch test reactions in the present study, although in different directions.

Although a biopsy from a positive patch test to this carpet, taken from a worker from Bank of Greenland, clearly showed spongiosis, indicating an allergic reaction, no final conclusions on allergic or irritant reactions can be drawn.

In conclusion, we find that it is predominantly likely that the reported symptoms are related to exposures from the building after renovation in 2009. A specific triggering exposure could – in spite of careful examination – not be identified, although chemicals from the glued carpets are suspected. Suspicion was drawn to *N*-methylsaccharin, being present in the carpet extracts with positive reactions only, but no definitive conclusion can be made. Our report confirms that finding the culprit – allergen or irritant – is a complicated process. The process includes a stepwise procedure, which was in the current case further complicated by the geographical location of the working place. The study illustrates the diversity of symptoms and exposures, which is often present in “Sick Building” cases. Dry climate in Greenland and dry indoor air in arctic buildings probably contribute to the high prevalence of symptoms.
